# Advanced Ankle and Foot Sonoanatomy: Imaging Beyond the Basics

**DOI:** 10.3390/diagnostics10030160

**Published:** 2020-03-14

**Authors:** Chen-Yu Hung, Ke-Vin Chang, Kamal Mezian, Ondřej Naňka, Wei-Ting Wu, Po-Cheng Hsu, Levent Özçakar

**Affiliations:** 1 Department of Physical Medicine and Rehabilitation, National Taiwan University Hospital, Bei-Hu Branch, 10845 Taipei, Taiwan; chenyu810@gmail.com (C.-Y.H.); wwtaustin@yahoo.com.tw (W.-T.W.); myronrbman@gmail.com (P.-C.H.); 2 Department of Physical Medicine and Rehabilitation, National Taiwan University College of Medicine, 10048 Taipei, Taiwan; 3 Department of Rehabilitation Medicine, Charles University, First Faculty of Medicine and General University Hospital in Prague, 12800 Prague, Czech Republic; kamal.mezian@gmail.com; 4 Institute of Anatomy, Charles University, First Faculty of Medicine, 12800 Prague, Czech Republic; ondrej.nanka@lf1.cuni.cz; 5 Department of Physical and Rehabilitation Medicine, Hacettepe University Medical School, 06100 Ankara, Turkey; lozcakar@yahoo.com

**Keywords:** ankle, foot, ultrasound, anatomy, pain

## Abstract

Ankle/foot pain is a common complaint encountered in clinical practice. Currently, due to the complex anatomy, the diagnosis and management of the underlying musculoskeletal disorders are extremely challenging. Nowadays, high-resolution ultrasound has emerged as the first-line tool to evaluate musculoskeletal disorders. There have been several existing protocols describing the fundamental sonoanatomy of ankle/foot joints. However, there are certain anatomic structures (e.g., Lisfranc ligament complex or Baxter nerve) which are also clinically important. As they are rarely elaborated in the available literature, a comprehensive review is necessary. In this regard, the present article aims to brief the regional anatomy, illustrate the scanning techniques, and emphasize the clinical relevance of the ankle/foot region.

## 1. Introduction

Ankle/foot pain is prevalent, affecting 15% to 24% of the adult population older than 45 years [1]. The complexity of the anatomy makes the diagnosis and management of the underlying musculoskeletal disorders extremely difficult. Herein, owing to its several advantages (e.g., portability, being free of radiation, real-time dynamic assessment, etc.), high-resolution ultrasound (US) has emerged as a useful tool in the diagnosis and treatment of musculoskeletal disorders [2,3]. There are several protocols elaborating the basic sonoanatomy of the ankle and foot, such as the anterior talofibular and calcaneofibular ligaments [4,5]. In recent years, more delicate but clinically significant anatomic structures have also been highlighted by cadaveric studies [6,7,8,9,10]. Until now, there is a lack of a comprehensive review elaborating the advances in US imaging techniques for the ankle and foot. In this sense, this article aims to elucidate the challenging anatomy, US scanning techniques, and clinical relevance of various structures/pathologies. All the images were obtained using the Aplio 300 machine (Canon, Japan) with a 4–14 MHz linear probe and the Logiq S8 machine (GE, USA) with a 4–15 MHz linear probe. The scanning depth was set at 2.5 cm for the superficial structure and 4 cm for the deep structure. The focus was adjusted at the same depth as our targets. The sensitivity of the power Doppler mode was tuned up to the most sensitive level with no flow signals detected under the bony cortex. Copious gel had been applied between the transducer and skin to avoid excessive compression on the targets. The settings were in accordance with our previous publications regarding US scanning protocols of the small parts [11,12]. The anatomy of the relevant structures was also elaborated using the cadaver models with the approval of the Anatomical Donation Department of Charles University in Prague. 

## 2. Ligaments

### 2.1. Bassett’s Ligament 

#### 2.1.1. Anatomy

**Figure 1:**
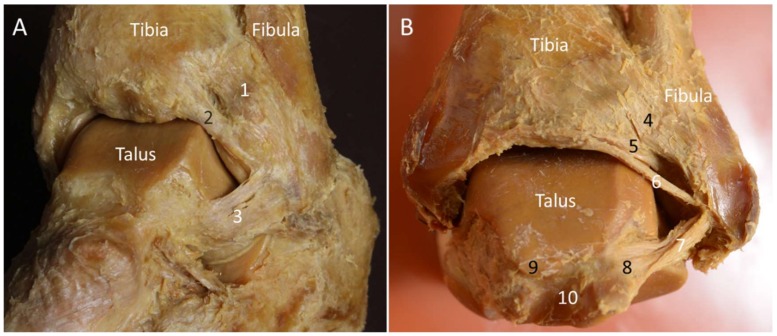
Lateral (**A**) and posterior (**B**) views of the ankle joint on the cadaveric model. 1: Anterior tibiofibular ligament; 2: Bassett’s ligament; 3: anterior talofibular ligament; 4: superficial component of the posterior tibiofibular ligament; 5: deep component of the posterior tibiofibular ligament; 6: intermalleolar ligament; 7: posterior talofibular ligament; 8: lateral tubercle of the posterior talar process; 9: medial tubercle of the posterior talar process; 10: tunnel for flexor hallucis longus.

The distal tibiofibular syndesmosis is a slightly movable fibrous joint whereby the tibia and fibula are linked and reinforced by the anterior and posterior inferior tibiofibular ligaments (AITFL and PITFL). The Bassett’s ligament (named after the physician who first reported it) is the most distal fascicle of the AITFL [13,14]. It courses inferolaterally from the anterior tubercle of the tibia to the distal fibula and lies deeper to the AITFL (Figure 1A). 

#### 2.1.2. Scanning Technique 

**Figure 2:**
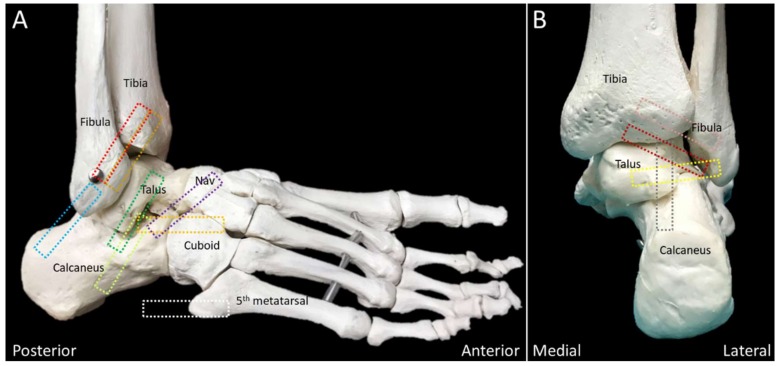
Colored dashed rectangles indicate the transducer positions through the lateral (**A**) and posterior (**B**) aspects of the ankle and foot. NAV, navicular.

**Figure 3:**
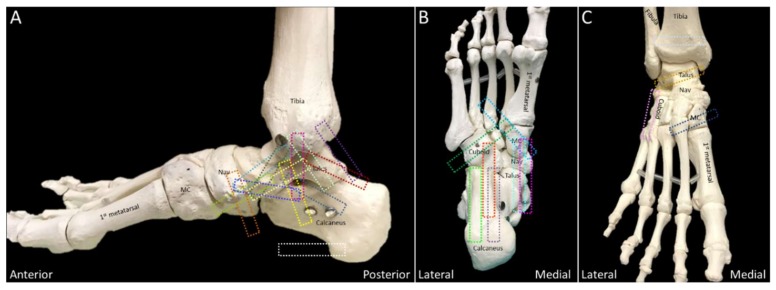
Colored dashed rectangles indicate the transducer positions on the medial (**A**), plantar (**B**), and anterior (**C**) aspects of the ankle and foot. MC: medial cuneiform; NAV: navicular.

Transducer positions for each anatomic structure are illustrated as the colored rectangles in Figure 2 and Figure 3. 

**Figure 4:**
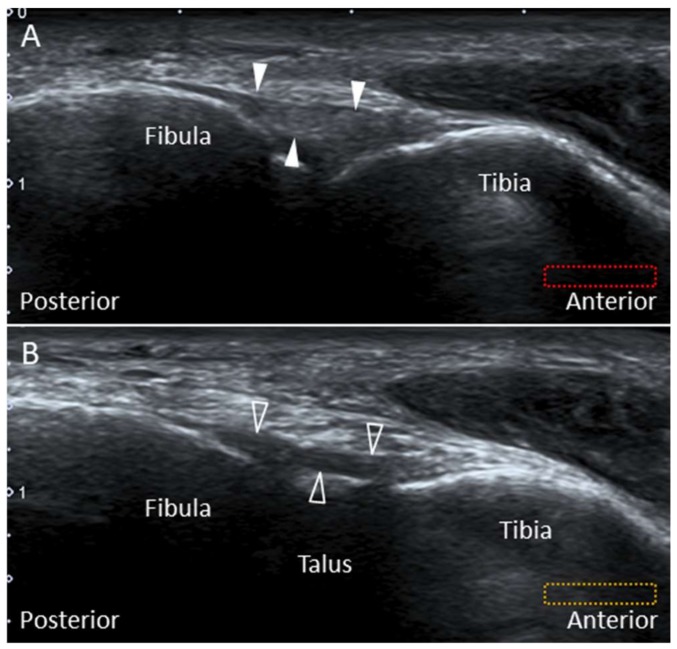
Imaging of the anterior-inferior tibiofibular (arrowheads) (**A**) and the Bassett’s (void arrowheads) ligaments (**B**). The different colored dashed rectangles are in accordance with the transducer positions in Figure 2A.

The transducer is initially placed in an oblique plane (proximal-medial to distal-lateral) at the distal syndesmosis to locate the AITFL (Figure 4A). Moving it distally, the Bassett’s ligament is seen deeper to the AITFL (Figure 4B). Of note, partial visualization of the bony contour of the talus provides a clue that the transducer is positioned on top of the Bassett’s ligament.

#### 2.1.3. Clinical Relevance

Normally, the talus rolls forward with simultaneous posterior sliding during ankle dorsiflexion. If the lateral ankle ligamentous complex is injured, this coupling movement disappears, leading to excessive anterior translation of the talus. A thickened Bassett’s ligament may be entrapped by the subluxed talus [13], and excision of this ligament can relieve the impingement without compromising the ankle joint stability [9]. 

### 2.2. Posterior Inferior Tibiofibular Ligament 

#### 2.2.1. Anatomy

The PITFL is composed of a superficial and a deep component (Figure 1B). The former originates from the posterior edge of the lateral malleolus and courses superomedially to the posterior tubercle of the tibia [15]. The deep portion—also named as the “inferior transverse ligament”—originates from the lateral malleolar fossa on the medial surface of the distal fibula and attaches to the posterior lateral edge of the distal tibia [15]. It has a cone shape and runs more horizontally than the superficial component, acting as a stabilizer for the ankle joint [15,16]. 

#### 2.2.2. Scanning Technique

**Figure 5:**
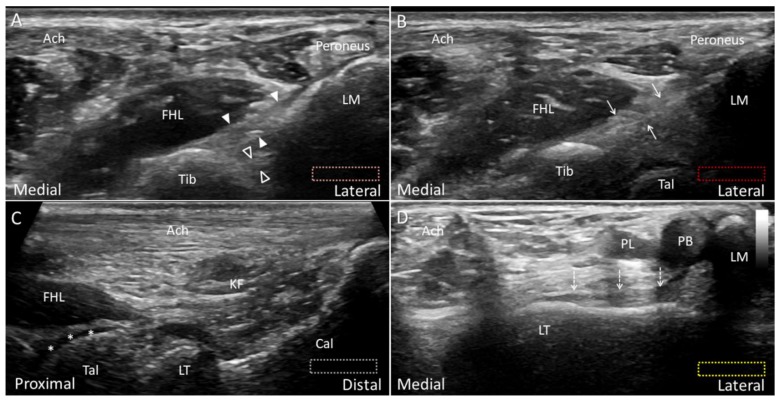
Ultrasound imaging of the posterior–inferior tibiofibular ligament (arrowheads, superficial component; void arrowheads, deep component) (**A**) and the intermalleolar ligament (arrows) (**B**). Placing the transducer along the long axis of the Achilles tendon (Ach), the lateral tubercle (LT) of the posterior talar process is visualized just cephalad to the posterior subtalar joint (**C**). Note the cartilage (asterisks) on the talar dome. As the lateral edge of the transducer is pivoted toward the lateral malleolus (LM), the posterior talofibular ligament (dotted arrows) is visualized lying on the talus (**D**). The different colored dashed rectangles are in accordance with the transducer positions in Figure 2B. Cal: calcaneus; FHL: flexor hallucis longus; KF: Karger’s fat pad; PB: peroneus brevis; PL: peroneus longus; Tib: tibia.

While the subject lies prone with the foot hanging out of the bed, the transducer is placed lateral to the Achilles tendon, at the level of the distal tibia, in the transverse oblique plane (proximal–medial to distal–lateral). Underneath the flexor hallucis longus muscle, the superficial part of the PITFL can be seen connecting the surface of the distal tibia and fibula. The deep portion travels underneath the superficial part with its lateral end hidden behind the acoustic shadow of the distal fibular tip (Figure 5A). 

#### 2.2.3. Clinical Relevance

Rupture of the PITFL may occur after excessive supination-eversion, pronation-eversion, or pronation-abduction of the ankle. As the PITFL is thick and strong, its injury usually results in avulsion of the bony attachment instead of rupture of the ligament’s middle portion [16]. The superficial and deep portions of the PITFL were reported to provide 9% and 33% (respectively) of the stability in the syndesmosis [16,17]. 

### 2.3. Intermalleolar Ligament 

#### 2.3.1. Anatomy

This ligament is located distal to the PITFL and proximal to the posterior talofibular ligament (PTFL). It courses superomedially from the fibula tip to the distal tibia—overlying the posterior talar dome (Figure 1A). The morphological features of this ligament are variable, depending on the sites of bony attachments, number of intra-ligamentous bundles, and the compactness of the bundle arrangement [7,15]. This ligament—found in almost all cadavers [18]—becomes tense during ankle dorsiflexion and loose at plantar flexion [15].

#### 2.3.2. Scanning Technique

The transducer is placed in the transverse oblique plane as the examination of PITFL and is moved distally. When the hypoechoic cartilage of the posterior talar dome is visualized, the intermalleolar ligament is seen superficial to the syndesmosis (Figure 5B). 

#### 2.3.3. Clinical Relevance

Forceful ankle dorsiflexion can lead to injury or even rupture of the intermalleolar ligament or osteochondral avulsion from its attachments [15]. After injury, the swollen ligament may cause impingement during plantar flexion as it is likely to be trapped between the distal tibia and fibula [15]. 

### 2.4. Posterior Talofibular Ligament (PTFL)

#### 2.4.1. Anatomy

Together with the anterior talofibular (ATFL) and calcaneofibular (CFL) ligaments, the PTFL constitutes the lateral collateral ligament complex. The PTFL is the strongest and deepest among the three aforementioned ligaments [9]. It originates from the lateral malleolar fossa and courses horizontally to attach on the lateral tubercle of the posterior talar process (Figure 1B) [15]. It is distal to the PITFL and intermalleolar ligament and is thicker than these two ligaments. The PTFL is tightened during ankle dorsiflexion and becomes relaxed at plantar flexion [15]. 

#### 2.4.2. Scanning Technique

The subject is positioned prone with the foot hanging outside of the examining bed. The ankle is dorsiflexed to tighten the PTFL. The transducer is aligned along the long axis of the Achilles tendon for identifying the lateral tubercle of the posterior talar process (Figure 5C). Then the transducer is rotated 90 degrees to bridge the lateral tubercle and distal lateral malleolus to see the PTFL superficial to the talus (Figure 5D). Unlike the PITFL and intermalleolar ligament, the PTFL is not located underneath the flexor hallucis muscle. 

#### 2.4.3. Clinical Relevance

The PTFL is seldom injured unless there is trauma with ankle dislocation. An isolated injury has never been reported in the pertinent literature [15]. 

### 2.5. Lateral Talocalcaneal Ligament

#### 2.5.1. Anatomy

**Figure 6:**
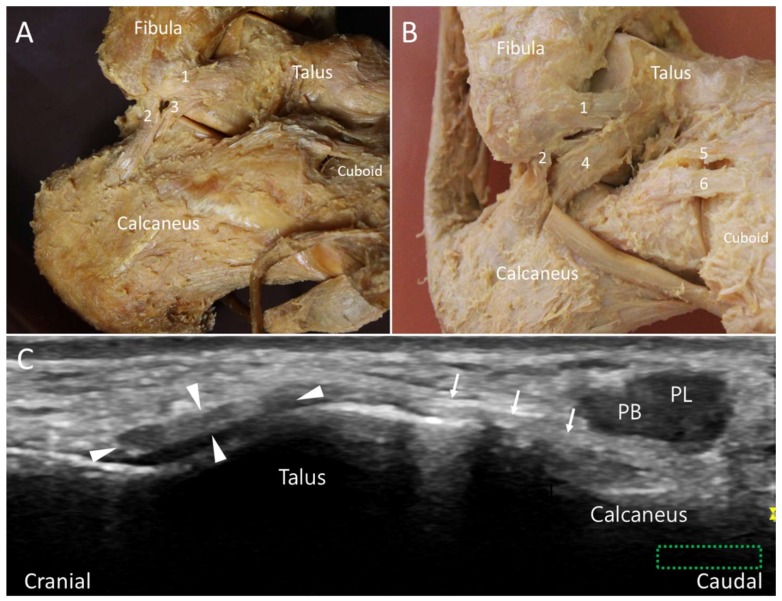
Type I (**A**) and type II (**B**) lateral talocalcaneal ligament in the cadaveric models. Ultrasound imaging shows the ligament (arrows) in its long axis (**C**). The colored dashed rectangle is in accordance with the transducer positions in Figure 2A. 1: Anterior talofibular ligament (arrowheads); 2: calcaneofibular ligament; 3: type I lateral talocalcaneal ligament; 4: type II lateral talocalcaneal ligament; 5: calcaneonavicular component of the bifurcate ligament; 6: calcaneocuboid component of the bifurcate ligament. PB: peroneus brevis tendon; PL: peroneus longus tendon.

The subtalar joint is stabilized by the CFL, lateral talocalcaneal ligament (LTCL), and sinus tarsi ligament. The last one can be further divided into the cervical and interosseous talocalcaneal ligaments. The LTCL may not be constantly present [9]. It runs parallel and anterior to the CFL, coursing from the lateral talus to the lateral calcaneus. It connects the ATFL on the talus [6] and forms a triangular complex with the ATFL and CFL to stabilize the lateral ankle and subtalar joints. The study by Trouilloud et al. classified the anatomy of LTCL into three types: type I, the LTCL branching from the CFL (Figure 6A); type II, the LTCL independent from the CFL (Figure 6B) and type III, absent LTCL [19]. In their cadaveric study, the incidence of type I, II, and III LTCL was 35%, 23%, and 42%, respectively. In another larger-sample-sized study, the LTCL was seen in 42% of the cadaveric specimens (type I 18% and type II 24%) [6]. Herewith, the study conducted by Jordi et al. found that the LTCL was always present as a component of the “lateral fibulotalocalcaneal ligament complex” [8]. 

#### 2.5.2. Scanning Technique

The examiner can first place the transducer at the distal fibula and direct it towards the big toe to locate the long axis of the ATFL. Afterward, the transducer is moved distally to visualize the talar attachment of the ATFL. The LTCL can be visualized by pivoting the anterior edge of the transducer toward the calcaneus (Figure 6C). The peroneus tendon complex can be seen on the surface of the LTCL. 

#### 2.5.3. Clinical Relevance

The LTCL helps prevent excessive supination, abduction/adduction of the subtalar joint [6]. Rupture of the LTCL has been implicated in chronic subtalar joint instability [20]. Its isolated tear is rare—otherwise associated with the CFL injury [21]. 

### 2.6. Bifurcate Ligament 

#### 2.6.1. Anatomy

**Figure 7:**
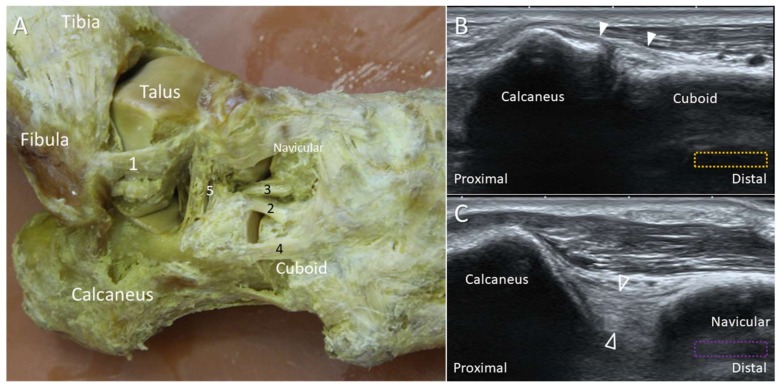
The cadaveric model (**A**) and ultrasound images (**B**,**C**) of the calcaneocuboid (white arrowheads) and calcaneonavicular (void arrowheads) ligaments. The colored dashed rectangles are in accordance with the transducer positions in Figure 2A. 1: Anterior talofibular ligament (ATFL); 2: calcaneocuboid ligament; 3: calcaneonavicular ligament (CNL); 4: lateral calcaneocuboid ligament; 5: cervical ligament.

The mid-tarsal, or Chopart joint, consists of the talonavicular and calcaneocuboid joints. The ligaments stabilizing this joint include the bifurcate, dorsal talonavicular, lateral calcaneocuboid, long plantar (LPL), and short plantar ligaments (SPL) [9]. The bifurcate ligament is a strong Y-shaped structure stabilizing the calcaneocuboid and talonavicular joints and has been described as the major stabilizer of the mid-tarsal joint [22]. It consists of two components: the calcaneonavicular (CNL) and calcaneocuboid (CCL) ligaments (Figure 7A). Both structures have proximal attachments from the anterior calcaneal process, and they course distally to attach on the navicular and cuboid bones, respectively [23]. The CNL appears like a cord, while the CCL is more likely to be a flattened band. On average, the CNL is twice as long and thick as the CCL [22]. The anatomy of the bifurcate ligament has been classified into three types: type I, presence of both components; type II, absence of the CCL; type III, absence of the CNL [24]. According to two cadaveric studies, the CNLs—but not the CCLs—were present in all specimens [22,24].

#### 2.6.2. Scanning Technique

With the subject’s foot resting on the table (foot flat position), the transducer is placed to bridge the calcaneocuboid joint. The CCL is viewed after the transducer is moved to reach the anterior calcaneal process (Figure 7B). For visualizing the CNL, the proximal pole of the transducer is fixed on the calcaneus while the distal pole is pivoted dorsally towards the navicular bone. The thick hyperechoic structure bridging the navicular and calcaneus is the CNL (Figure 7C). 

#### 2.6.3. Clinical Relevance

Mid-tarsal sprain is derived from ligamentous injuries whereby the bifurcate and lateral CCL are commonly affected. An avulsion fracture of the anterior calcaneal process at the origin of the bifurcate ligament should be considered in patients with severe ankle inversional injuries [23]. 

### 2.7. Deltoid Ligament 

#### 2.7.1. Anatomy

**Figure 8:**
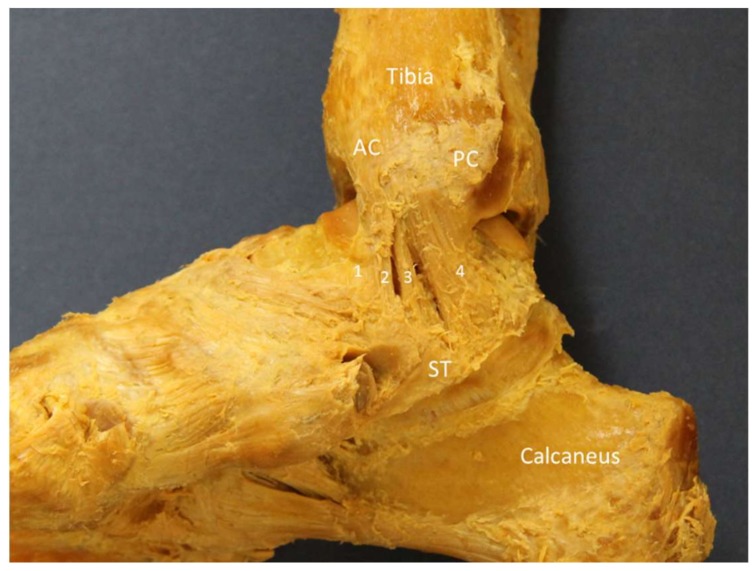
The cadaveric model of the deltoid ligament. 1: tibionavicular ligament; 2: tibiospring ligament; 3: tibiocalcaneal ligament; 4: posterior tibiotalar ligament; AC: anterior colliculus; PC: posterior colliculus; ST: sustentaculum tali.

There are considerable anatomic variations of the deltoid ligament. Besides, the components of the ligamentous complex are connected to each other instead of being separate. The deltoid ligament is divided into the superficial and deep layers. In the former one, the tibionavicular ligament originates from the anterior colliculus (the anterior portion of the medial malleolus) and attaches onto the navicular bone. The tibiospring ligament (the most superficial band of the deltoid ligament) has a similar proximal origin as the tibionavicular ligament and attaches to the superomedial component of the spring ligament. The tibiocalcaneal ligament originates from the intercollicular groove of the medial malleolus and attaches to the sustentaculum tali. The superficial posterior tibiotalar ligament also originates from the intercollicular groove and attaches onto the posterior talar body (Figure 8). 

In the deep layer, the anterior tibiotalar ligament originates from the anterior colliculus and attaches to the medial talus. It is usually thin and not always present. It lies deep to the tibionavicular and tibiospring ligaments. The deep posterior tibiotalar ligament—the thickest band of the deltoid ligament—originates from the intercollicular groove and attaches to the posterosuperior talus.

#### 2.7.2. Scanning Technique

**Figure 9:**
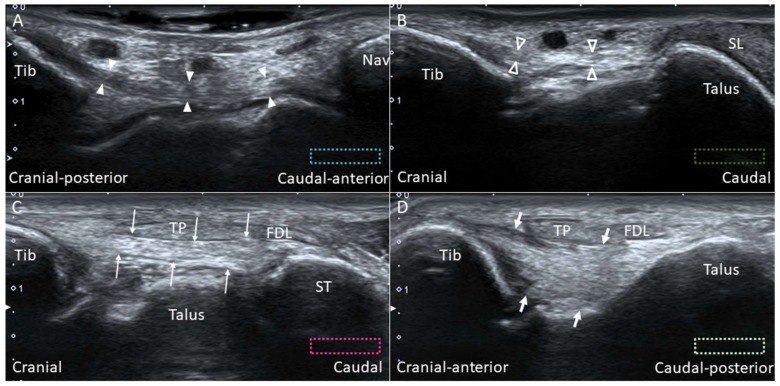
Imaging for the tibionavicular (white arrowheads) (**A**), tibiospring (void arrowheads) (**B**), tibiocalcaneal (thin arrows) (**C**) and tibiotalar (thick arrows) (**D**) ligaments. The colored dashed rectangles are in accordance with the transducer positions in Figure 3A. FDL: flexor digitorum longus tendon; Nav: navicular; SL: spring ligament; ST: sustentaculum tali; Tib: tibia; TP: tibialis posterior tendon.

Scanning of each component of the deltoid ligament can be facilitated by palpating the anterior and posterior colliculi. The ankle can be everted to tighten the deltoid ligament. The transducer is placed over the anterior colliculus and directed towards the navicular and talus to visualize the tibionavicular (Figure 9A) and tibiospring (Figure 9B) ligaments. These two ligaments are thin, band-like structures, with the tibialis posterior (TP) tendon lying superficial to them. The anterior tibiotalar ligament also originates from the anterior colliculus, but it is difficult to visualize it under US. The transducer is first positioned over the inter-collicular groove and then pivoted towards the sustentaculum tali and the posterior talar body to visualize the tibiocalcaneal (Figure 9C) and posterior tibiotalar (Figure 9D) ligaments.

#### 2.7.3. Clinical Relevance

With the use of high-resolution US, the incidence of deltoid ligament injury is realized to be higher than previously thought [25]. It was reported that the deltoid ligament is involved in approximately 15% of all ligamentous injuries [26]. The pertinent mechanism of injury is either excessive pronation or supination-external rotation of the ankle [27]. In the latter mechanism (which is common in ankle inversional sprain), the lateral ankle ligamentous complex may be simultaneously disrupted, leading to multidirectional ankle instability. 

### 2.8. Spring Ligament 

#### 2.8.1. Anatomy

**Figure 10:**
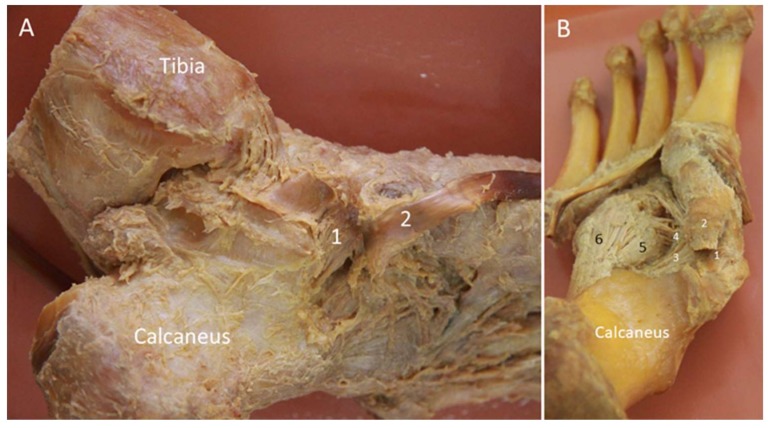
The cadaveric model for the spring ligament viewed from the medial (**A**) and plantar (**B**) aspects. 1: Superomedial component; 2: tibialis posterior tendon; 3: medioplantar component; 4: inferoplantar component; 5: short plantar ligament; 6: long plantar ligament.

The spring ligament was initially considered to be elastic, acting as a spring to support the medial longitudinal foot arch. However, subsequent histologic studies revealed that the spring ligament is purely collagenous without elastic fibers [28,29,30]. Despite this, the term “spring ligament” is still widely used. The ligament has three components: the superomedial, medioplantar, and inferoplantar ligaments (Figure 10). The superomedial component originates from the medial aspect of the sustentaculum tali and attaches to the superomedial aspect of the navicular near the talonavicular joint [9]. The medioplantar component originates from the coronoid fossa at the anterior aspect of calcaneus and attaches just below the navicular tuberosity. The inferoplantar component originates from the coronoid fossa anterior to the origin of the medioplantar component and inserts on the inferior beak of the navicular [28]. Among the three, the superomedial ligament is the broadest and clinically the most important one. It functions as a sling underneath the talus. 

#### 2.8.2. Scanning Technique

**Figure 11:**
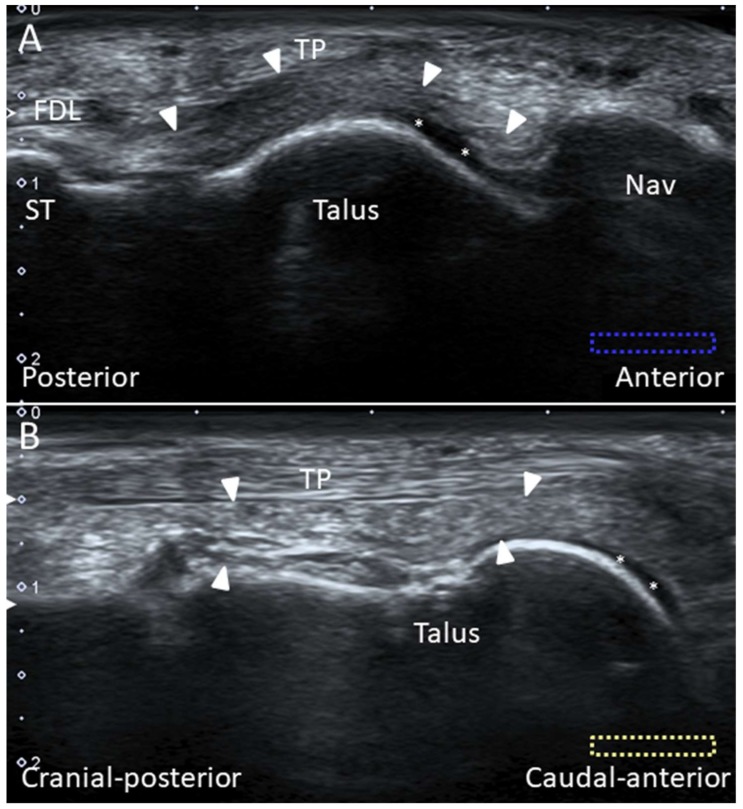
Imaging of the superomedial component of the spring ligament (arrowheads) (**A**). The spring ligament is in the oblique-short axis underneath the long axis of the tibialis posterior tendon (**B**). Please note the cartilage (asterisks) on the distal talus. The colored dashed rectangles are in accordance with the transducer positions in Figure 3A. FDL: flexor digitorum longus tendon; Nav: navicular; ST: sustentaculum tali.

While the superomedial ligament can easily be depicted using US, it is difficult to visualize the other two components (due to their deep positions). For examination of the superomedial ligament, the probe is placed parallel to its long axis, with the proximal end placed over the sustentaculum tali and the distal end pivoted dorsally toward the talus and the superomedial aspect of the navicular. The ligament can be visualized between the TP tendon superficially and the talus deeply (Figure 11A). The ligament is the widest in its proximal portion [31]. There is a crisscross between the TP tendon and the spring ligament (Figure 11B). 

#### 2.8.3. Clinical Relevance

An isolated spring ligament injury is uncommon, and can be found in 70% of cases with some degree of deformity secondary to TP tendon insufficiency [28]. The spring ligament injury is more common at the superomedial portion. A loose spring ligament results in valgus alignment of the calcaneus and flat foot deformity [31]. 

### 2.9. Plantar Ligament

#### 2.9.1. Anatomy

**Figure 12:**
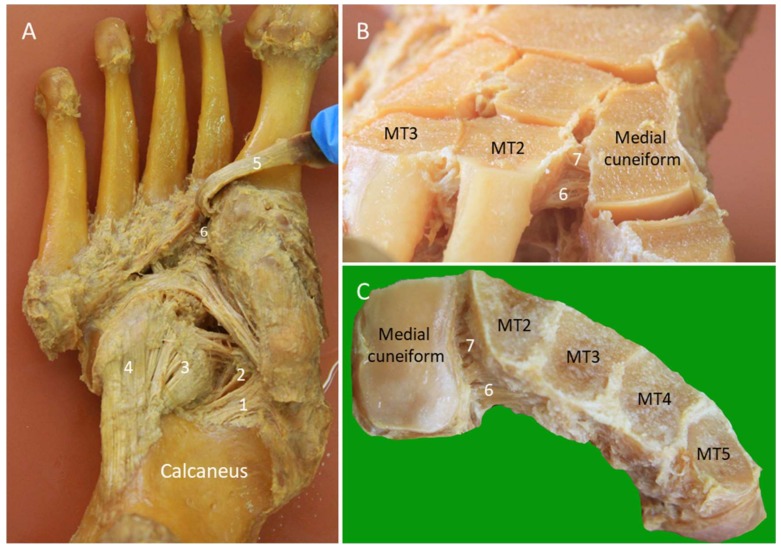
Cadaveric models of the long and short plantar ligaments (**A**). Dorsal (**B**) and axial (**C**) transections of the Lisfranc ligament complex. 1: Medioplantar component of the spring ligament; 2: inferoplantar component of the spring ligament; 3: short plantar ligament; 4: long plantar ligament; 5: peroneus longus tendon; 6: plantar Lisfranc ligament; 7: interosseous Lisfranc ligament; MT: meta-tarsal bone.

The LPL originates from the plantar surface between the calcaneal tuberosity and the anterior calcaneal tubercle. It attaches to the second to fifth metatarsal bases, cuboid tuberosity, and plantar surface of the cuboid [9]. The SPL or the plantar calcaneocuboid ligament lies deeper and more medial than the LPL. The SPL is separated from the LPL by a layer of loose areolar tissue. It originates from the anterior calcaneal tubercle and inserts on the plantar surface of the cuboid, proximal to the cuboid tunnel where the PL tendon traverses. It is short but strong and provides strong support to the lateral foot arch [9] (Figure 12A). 

#### 2.9.2. Scanning Technique

**Figure 13:**
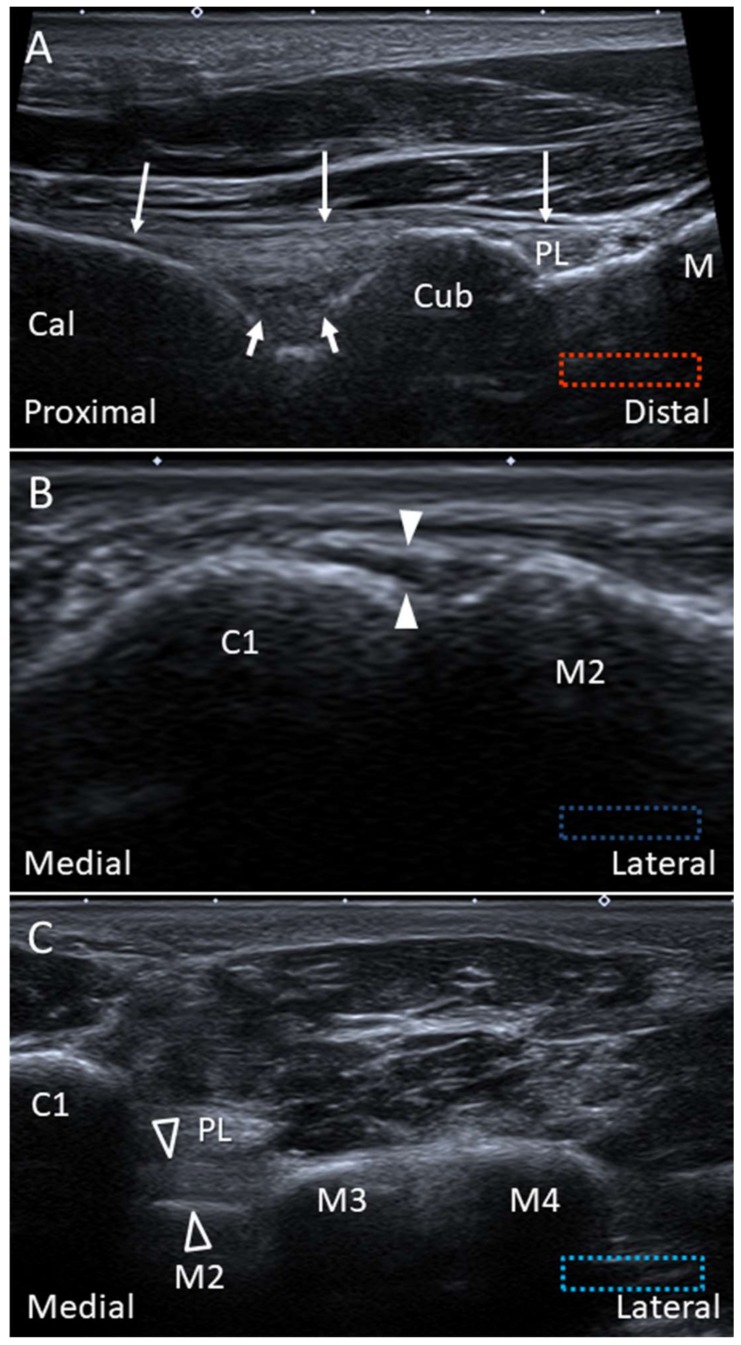
Imaging of the long (thin arrows) and short plantar (thick arrows) ligaments (**A**), dorsal (white arrowheads) (**B**) and plantar (void arrowheads) (**C**) Lisfranc ligaments. Cal: calcaneus; Cub: cuboid; C1: medial cuneiform; M2: 2nd metatarsal bone: M3: 3rd metatarsal bone; M4: 4th metatarsal bone; PL: peroneus longus.

The transducer is placed on the plantar hind foot to visualize the LPL and SPL. The peroneus longus (PL) tendon, coursing anterior to the cuboid tuberosity, can serve as an important landmark. The LPL runs above the PL tendon and is seen attached to the metatarsal bases. Dynamic examination by dorsiflexion of the toes can help evaluate the LPL integrity. The SPL is seen deep to the LPL across the calcaneocuboid joint, and the differentiation between the two ligaments may not be easy (Figure 13A). 

#### 2.9.3. Clinical Relevance

The LPL and SPL can be involved in both inversion and eversion types of mid-tarsal sprain. Their injury is less frequent than the other ligaments over the mid-tarsal joints [23]. 

### 2.10. Lisfranc Ligament Complex

#### 2.10.1. Anatomy

The Lisfranc joint complex bridges the tarso-metatarsal articulations (the junctions between the fore- and mid-foot). It courses obliquely from the lateral aspect of the medial cuneiform to the medial aspect of the second metatarsal base. It has dorsal, interosseous, and plantar components. The dorsal component is the thinnest and is therefore vulnerable to injury (Figure 12B,C). While the second to fifth metatarsals are stabilized by the intermetatarsal ligaments, the space between the first and second metatarsals is stabilized by the Lisfranc ligament complex instead.

#### 2.10.2. Scanning Technique

The foot is placed on the examination bed for scanning the dorsal Lisfranc ligament. The transducer is put between the first and second metatarsal shafts along the axial plane and is then moved proximally until the first tarsometatarsal joint is seen. Moving the transducer further proximally, the dorsal surface of the medial cuneiform is visualized as an angulated bony cortex. In contrast, the second metatarsal has a rounded dorsal bony surface. The transducer is gently adjusted until the dorsal Lisfranc ligament is identified (Figure 13B). Thickening, hypoechogenicity, or absence of the ligament indicate tears of the dorsal Lisfranc ligament. Widening of the space between the medial cuneiform and second metatarsal may be seen. The space can be further increased by weight-bearing [32] or manual compression of either the medial cuneiform or the second metatarsal by the examiner (Supplementary Video S1). 

The plantar Lisfranc ligament is perpendicular to the course of the PL tendon on the plantar foot. US scanning can start by identifying the long axis of the PL tendon attachment. Later, the transducer is rotated 90 degrees to reach the medial cuneiform. The plantar Lisfranc ligament is visualized as a thick fibrillar structure connecting the medial cuneiform and the second metatarsal base, with the PL tendon lying superficial to it (Figure 13C). The extension of the plantar Lisfranc ligament to the third metatarsal base can also be visualized.

#### 2.10.3. Clinical Relevance

The incidence of Lisfranc ligament injury is rare, accounting for approximately 0.2% of all cases with foot fractures whereby up to 20% of the cases have delayed diagnosis [33]. However, accurate diagnosis is quite difficult [33]. In patients with acute mid-foot pain after trauma, clinicians should look for the possibility of a complete rupture of the Lisfranc ligament as the lesion may necessitate surgical repair for foot instability [34]. 

### 2.11. Lateral Cord of the Plantar Fascia

#### 2.11.1. Anatomy

**Figure 14:**
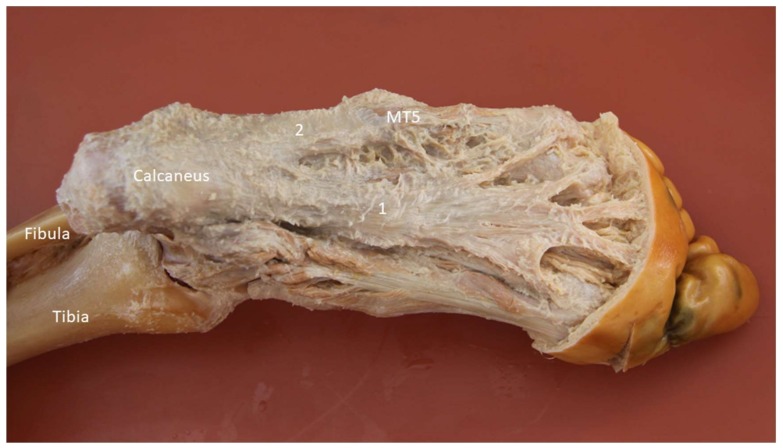
Cadaveric model of the lateral cord of plantar fascia (2). 1: Medial cord of plantar fascia; MT5: fifth metatarsal base.

The lateral cord of the plantar fascia (LCPF) arises from the lateral aspect of the medial calcaneal tuberosity where it blends partially with the origin of the underlying abductor digiti minimi muscle and the lateral aspect of the central cord of the plantar fascia [35]. Distally, it courses towards the base of the fifth metatarsal bone and is superficial to the abductor digiti minimi muscle (Figure 14). The main portion of the LCPF finally attaches on the lateral tuberosity of the fifth metatarsal base with a broad footprint ventral to the peroneus brevis (PB) tendon insertion.

#### 2.11.2. Scanning Technique

**Figure 15:**
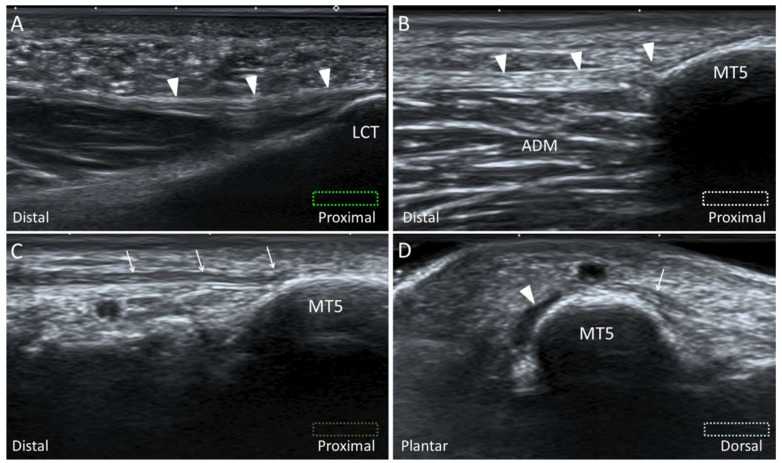
Imaging of the lateral cord (arrowheads) of the plantar fascia at the proximal origin (**A**) and distal attachment (**B**). Moving the transducer dorsally, the peroneus brevis tendon (arrows) is also visualized attaching on the fifth metatarsal base (MT5) (**C**). The short axis imaging demonstrates the anatomic relationships between the lateral cord of plantar fascia and the peroneus brevis tendon (**D**). The colored dashed rectangles are in accordance with the transducer positions in Figure 2A and Figure 3B. ADM: abductor digiti minimi; LCT: lateral calcaneal tuberosity..

For examination of the proximal and mid portions of the LCPF, the transducer is placed along the sagittal plane on the lateral plantar surface (Figure 15A) [35]. For examination of the distal LCPF (the most common place for pathologies), the transducer is placed on the lateral aspect of the fifth metatarsal base. The LCPF is seen as a widening insertion with a hyperechoic fibrillar pattern (Figure 15B). The abductor digiti minimi muscle is located dorsal to the LCPF. Moving the transducer slightly to the dorsal aspect, the PB tendon is seen as a smaller footprint on the dorsomedial aspect of the fifth metatarsal base (Figure 15C,D) [35].

#### 2.11.3. Clinical Relevance

Stress fracture of the fifth metatarsal base, cuboid, or os peroneum, tendinopathy of the PL and PB tendons, and strain of the abductor digiti minimi muscle are common causes of lateral mid-foot pain [36]. Recently, the LCPF has been found to be another important pain generator [35,37]. Since the aforementioned pathologies may be overlapped, US serves as a critical tool for precise diagnosis. 

## 3. Retinacula

### 3.1. Anatomy

**Figure 16:**
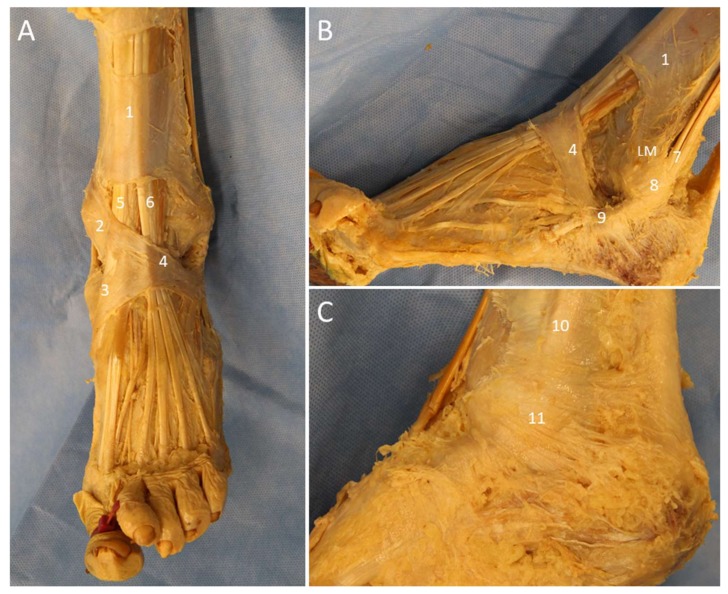
Cadaveric models of the extensor (**A**), peroneal (**B**), and flexor (**C**) retinacula. 1: superior extensor retinaculum; 2: oblique superomedial band of the inferior extensor retinaculum; 3: oblique inferomedial band of the inferior retinaculum; 4: frondiform ligament; 5: tibialis anterior tendon; 6: extensor digitorum longus; 7: peroneus longus and brevis tendons; 8: superior peroneal retinaculum; 9: inferior peroneal retinaculum; 10: tibialis posterior tendon; 11: flexor retinaculum; LM: lateral malleolus.

The ankle retinaculum is the focal thickening of the crural fascia that stabilizes the tendons to prevent bowstringing during muscle contractions [38]. The retinacula of the ankle and foot comprise the superior and inferior extensor retinacula (SER and IER) (Figure 16A), the superior and inferior peroneal retinacula (SPR and IPR) (Figure 16B), and the flexor retinaculum (FR) (Figure 16C). 

The SER is a transverse rectangular band proximal to the ankle joint. It attaches medially on the anterior crest of the tibia and medial malleolus and laterally on the lateral crest of the fibula and the lateral malleolus [38,39]. From medial to lateral, it covers the tibialis anterior, extensor hallucis longus, extensor digitorum longus, and peroneus tertius tendons. A separate tunnel for the tibialis anterior tendon is formed by the superficial and deep fibers in 25% of cases [39]. 

The IER is a Y-shaped structure comprising the frondiform ligament and the oblique superomedial and inferomedial bands. The oblique superolateral band is present in various percentages of cases (25–81%) and, when present, shapes the IER into the cruciform configuration [39]. The frondiform ligament is the main part of the IER, retaining the extensor digitorum longus and peroneus tertius (PT) tendons against the talus and calcaneus. It has three roots attaching into the sinus tarsi. The oblique superomedial band continues with the frondiform ligament to attach on the medial malleolus. It covers the extensor hallucis longus tendon and separates into two fascicles surrounding the tibialis anterior tendon. The oblique inferomedial band arises from the apex of the frondiform ligament and is directed towards the naviculocuneiform joint. The oblique superolateral band, if present, is usually the thinnest and crosses over the AITFL to insert on the lateral malleolus [38,39]. 

The SPR originates from the periosteum of the lateral border of the retro-malleolar groove and the tip of lateral malleolus and inserts onto the lateral wall of the calcaneus. At the fibular insertion of the SPR, a small triangular fibrocartilage is frequently present which functions to deepen the concavity of the groove. The IPR is the lateral continuation of the IER and attaches to the inferolateral aspect of the calcaneus. It has deep fibers attaching to the trochlear process of the calcaneus, which separate the two fibro-osseous tunnels for the passage of the PL and PB tendons [40]. 

The FR is a triangular fibrous band originating from the tip of medial malleolus and extending to the posterosuperior aspect of the medial calcaneus. It forms the roof of the tarsal tunnel. Within the tunnel, TP, flexor digitorum longus, and flexor hallucis tendons are compartmented by fibrous septa from the FR [39]. 

### 3.2. Scanning Technique

**Figure 17:**
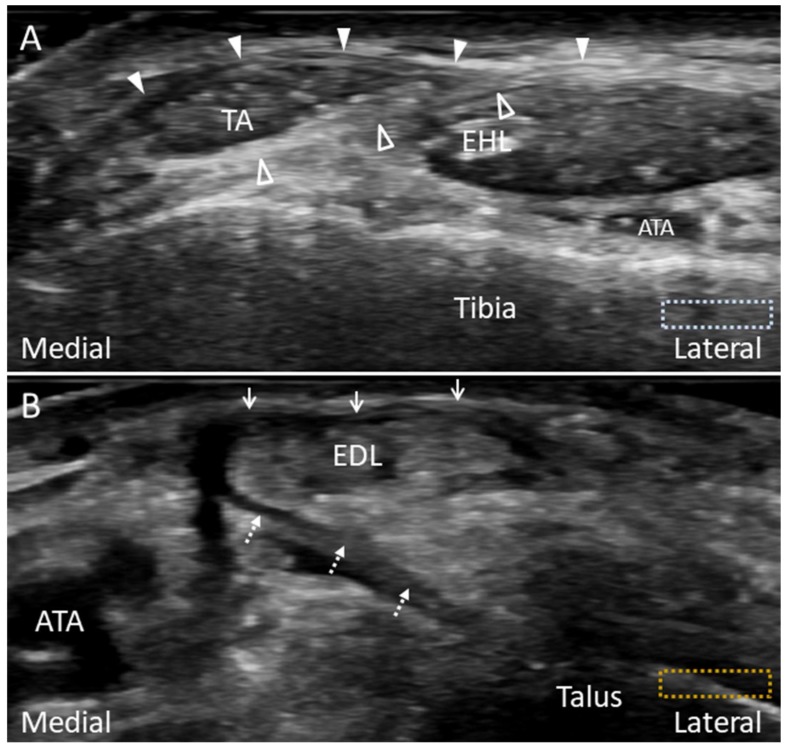
Imaging of the superior extensor retinaculum (**A**) and frondiform ligament of the inferior extensor retinaculum (**B**). The superior extensor retinaculum divides into the superficial (arrowheads) and deep (void arrowheads) layers, forming a separate tunnel for the tibialis anterior tendon (TA) (**A**). The lateral (arrows) and medial (dotted arrows) roots of the frondiform ligament can be seen attaching to the sinus tarsi (**B**). The colored dashed rectangles are in accordance with the transducer positions in Figure 3C. ATA: anterior tibial artery; EHL: extensor hallucis longus; EDL: extensor digitorum longus.

The retinacula are better evaluated in the long axis, while the underlying tendons are better scanned in the short axis. Under US imaging, the normal retinacula appear as thin (approximately 1 mm) hyperechoic fibrillar bands [39]. Injury of the retinacula may result in thickening, complete rupture, or avulsion. For evaluation of the SER and IER, the transducer is placed proximal to and on the anterior ankle joint, respectively. The divisions of the SER into the superficial and the deep layers can be observed above the tibialis anterior tendon (Figure 17A). The frondiform ligament of the IER and its roots are best assessed on the coronal oblique plane with the probe placed towards the sinus tarsi (Figure 17B) [38]. 

**Figure 18:**
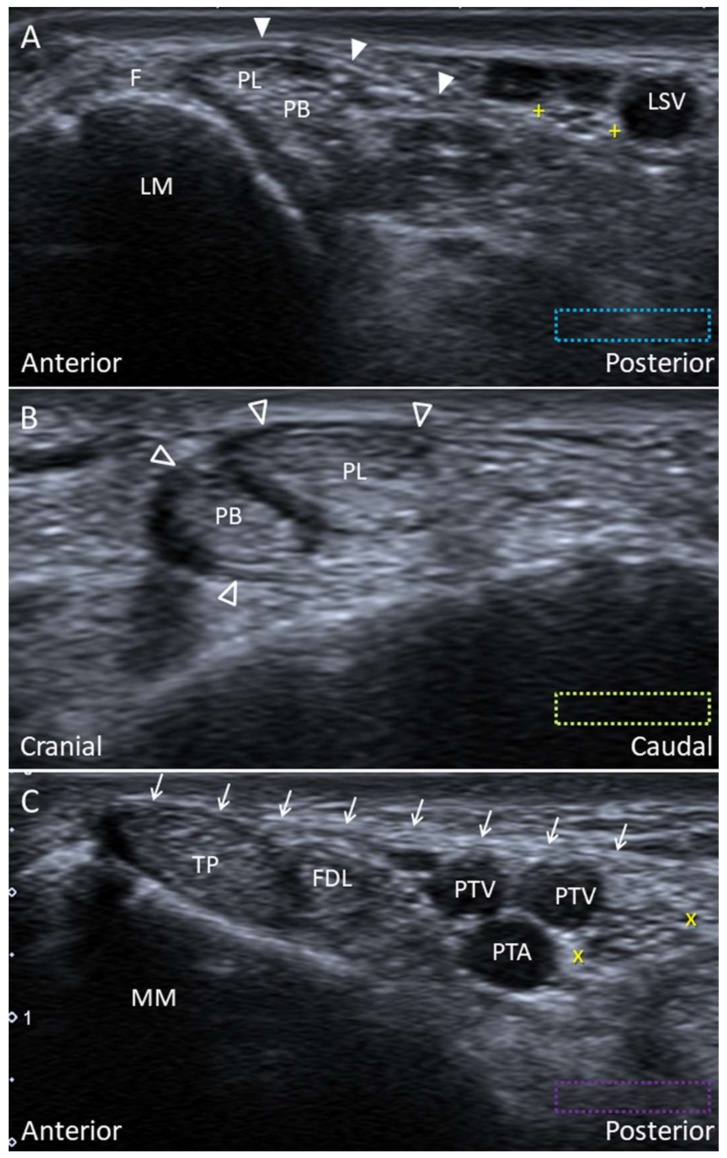
Imaging of the superior (arrowhead) (**A**) and inferior peroneal (void arrowheads) (**B**) retinacula and the flexor retinaculum (arrows) (**C**). The colored dashed rectangles are in accordance with the transducer positions in Figure 2A and Figure 3A. “+ +”: lateral sural nerve; “x x”: tibial nerve; F: fibrocartilage; FDL: flexor digitorum longus tendon; LM: lateral malleolus; LSV: lesser saphenous vein; MM: medial malleolus; PB: peroneus brevis tendon; PL: peroneus longus tendon; PTA: posterior tibial artery; PTV: posterior tibial vein; TP: tibialis posterior tendon.

The SPR and IPR are evaluated by placing the probe along their long axes (Figure 18A,B). In SPR injuries, the complex of the triangular fibrocartilage on the fibula and the peroneal tendons should also be evaluated for stability by asking the patients to forcefully dorsiflex and evert their ankles (). Unstable peroneal tendons may be subluxed anterior to the peroneal groove or onto the tip of the lateral malleolus secondary to the stripping injury, avulsion or rupture of the SPR [41]. The FR is depicted with the transducer placed along the axial oblique plane posterior to the medial malleolus (Figure 18C). Dynamic examination with active ankle dorsiflexion and inversion can also be performed for examining the stability of the TP tendon [39]. 

### 3.3. Clinical Relevance

Injury of the retinacula is common in patients with ankle pain and usually develops after ankle sprain. The clinicians should routinely evaluate the ankle retinacula in patients suffering ankle/foot trauma by static/dynamic US imaging [38].

## 4. Tendons 

### 4.1. Peroneus Longus Tendon

#### 4.1.1. Anatomy

**Figure 19:**
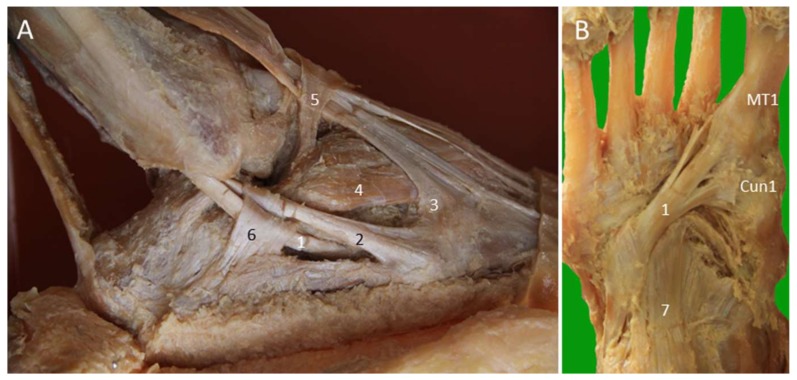
Cadaveric models for the peroneal tendons at the lateral ankle (**A**) and the peroneus longus tendon at the plantar aspect (**B**). 1: Peroneus longus tendon; 2: peroneus brevis tendon; 3: peroneus tertius tendon; 4: extensor digitorum brevis muscle; 5: frondiform ligament of the inferior extensor retinaculum; 6: inferior peroneal retinaculum; 7: long plantar ligament. Cun1: medial cuneiform; MT1: first metatarsal base.

The PL tendon is long and changes its course at three fibro-osseous tunnels: at the lateral malleolus within the retro-malleolar groove bounded posteriorly by the SPR (Figure 16B); at the peroneal tubercle, along the lateral wall of calcaneus and encircled by the IPR (Figure 19A); and at the cuboid tuberosity underneath the LPL (Figure 19B). The cuboid tuberosity serves as the proximal-medial wall of the cuboid tunnel, a shallow and obliquely oriented indentation of the plantar cuboid surface [42]. The PL tendon traverses the cuboid tunnel and inserts mainly on the first metatarsal base. Other insertions of the distal PL tendon include the medial cuneiform, plantar second to fifth metatarsals, and the first dorsal interosseous muscle [43]. The distal PL tendon helps to stabilize the first tarsometatarsal joint and also aids in the plantar flexion of the foot.

#### 4.1.2. Scanning Technique

**Figure 20:**
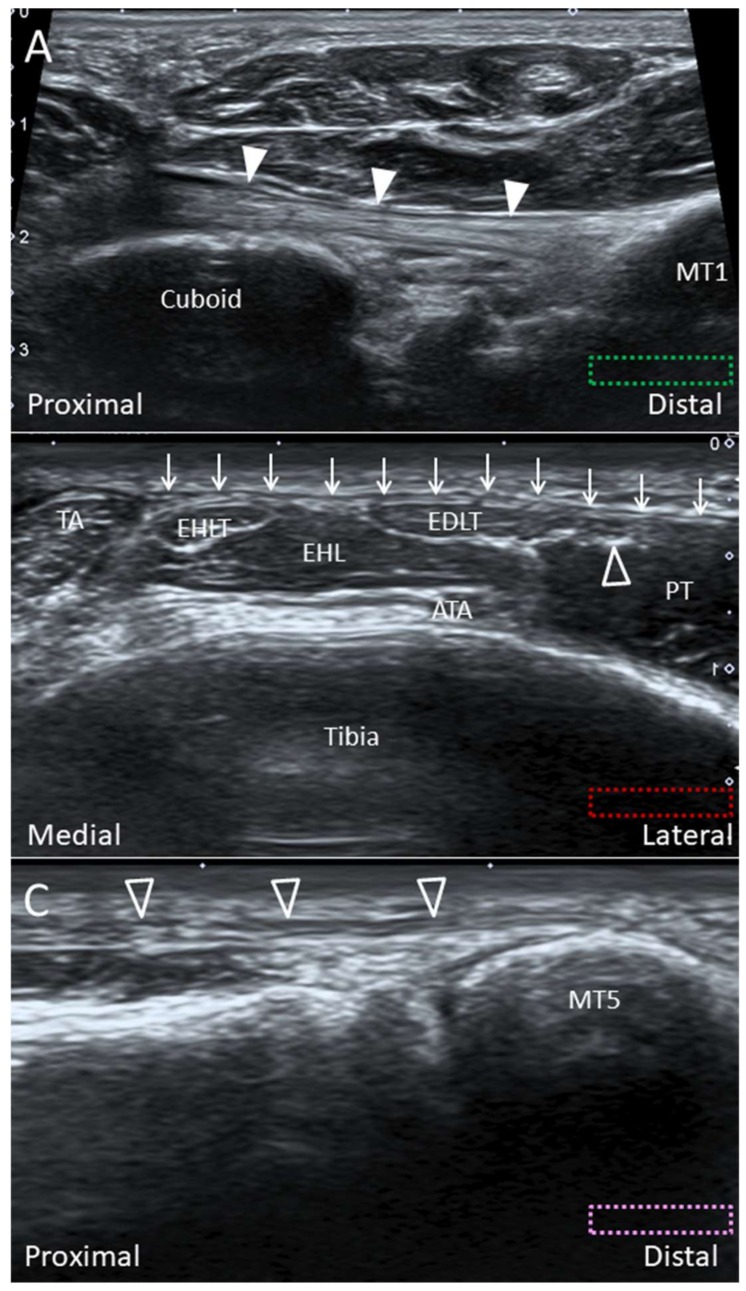
Imaging of the peroneus longus tendon (white arrowheads) in the long axis at the distal insertion (**A**), the peroneus tertius tendon (void arrowhead) in short axis at the distal tibia level (**B**)**,** and in long axis at the distal insertion (**C**). The colored dashed rectangles are in accordance with the transducer positions in Figure 3B,C. Arrows: superior extensor retinaculum; ATA: anterior tibial artery; EHL: extensor hallucis longus muscle; EHLT: extensor hallucis longus tendon; FDLT: flexor digitorum longus tendon; MT: metatarsal bone; PT: peroneus tertius muscle; TA: tibialis anterior tendon.

For scanning the PL tendon on the plantar surface, the transducer is placed along the sagittal plane of the plantar foot. The tendon is identified at its oblique axis distal to the cuboid tuberosity (Figure 13A). It might be seen subluxated outside of the cuboid tunnel in some cases during ankle dorsiflexion, even in asymptomatic subjects [42]. The transducer is then realigned to the long axis of the tendon towards the distal insertion onto the first metatarsal base (Figure 20A). Adjacent plantar vessels are frequently seen near the tendon and should not be misinterpreted as peritendinous effusion. 

#### 4.1.3. Clinical Relevance

The pathologies of the proximal PL tendon usually cause pain over the distal fibula tip or the peroneal tubercle. On the contrary, injuries of the distal tendon insertion would be manifested as medial mid-foot pain. Although uncommon, the disorders of the distal PL tendon include tendinosis, tears and avulsion of the first metatarsal base [44].

### 4.2. Peroneus Tertius Tendon

#### 4.2.1. Anatomy

The PT muscle originates from the distal one-third of the fibula shaft and the interosseous membrane [45]. After leaving the undersurface of the SER and IER, the PT tendon courses just lateral to the extensor digitorum longus tendons, and it has a broad insertion on the superior aspect of the fifth metatarsal base (Figure 19A). The PT is often considered as a part of the extensor digitorum longus muscle and its fifth tendon [46]. The presence of this muscle has been reported to range between 88.2% to 100% [45]. 

#### 4.2.2. Scanning Technique

The transducer is placed in the transverse plane at the level of the distal tibia. Short-axis imaging for tibialis anterior, extensor hallucis longus, extensor digitorum longus, and PT tendons can be performed from medial to lateral (Figure 20B). Lateral to the extensor digitorum longus and PT tendons would lie the PT muscle. The long axis of the PT tendon can be visualized as a fibrillar structure by pivoting the transducer towards the fifth metatarsal base (Figure 20C). 

#### 4.2.3. Clinical Relevance

The study by Witvrouw et al. found that the function of the PT can be considered as fine-tuning of the foot position during the swing phase rather than as a major contributor in the eversion/dorsiflexion strength [47]. While the injury of PT is rare, tendon tear and snapping due to hypertrophied tendon/muscle have been reported [46,48]. 

### 4.3. Tibialis Posterior Tendon

#### 4.3.1. Anatomy

After curving around the medial malleolus, the TP tendon divides into the anterior, middle, and posterior components just proximal to the navicular. It has multiple insertions on the hindfoot (except the talus), midfoot, and the base of the forefoot (Figure 11A,B) [49]. The anterior component, also called the direct tendon, is the largest portion and inserts onto the navicular and medial cuneiform. The middle component, also called the tarsometatarsal tendon, inserts onto the intermediate and lateral cuneiforms, cuboid, second to fourth metatarsal bases, PL, and flexor hallucis brevis tendons [50]. The posterior component arises from the main tendon proximal to the navicular and curves backward to insert on the anterior aspect of the sustentaculum tali [49].

#### 4.3.2. Scanning Technique

**Figure 21:**
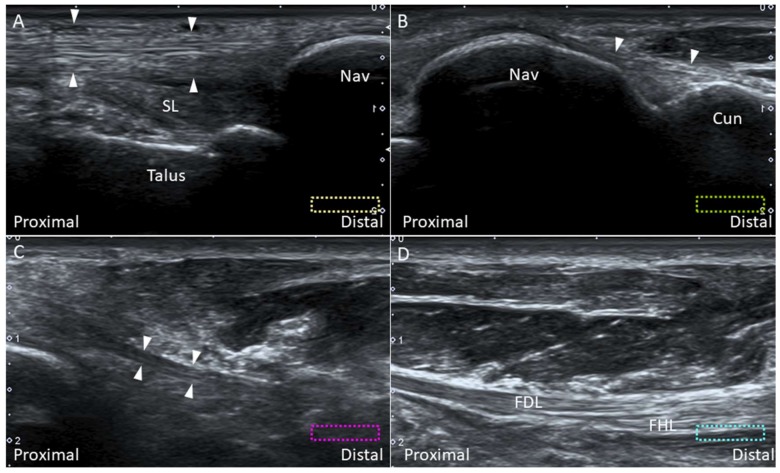
Imaging of the insertion of the direct limb of the tibialis posterior tendon (arrowheads) on the navicular (**A**) and medial cuneiform (**B**) bones and the tarsometatarsal portion of the tibialis posterior tendon (**C**). Moving the transducer to the plantar aspect of the sole, the knot of Henry can be seen (**D**). The colored dashed rectangles are in accordance with the transducer positions in Figure 3A,B. Cun: medial cuneiform; FDL: flexor digitorum longus tendon; FHL: flexor hallucis longus tendon; Nav: navicular; SL: spring ligament.

To examine the direct TP tendon, the transducer is aligned with the long axis of the tendon from the medial retromalleolar groove to the navicular tubercle. Accessory navicular bones may be identified in some cases (Figure 21A). The transducer is then moved slightly to the plantar surface and tilted upward to visualize the insertion on the medial cuneiform (Figure 21B). To visualize the tarsometatarsal component of the tendon, the transducer is moved more inferiorly to follow it running under the abductor hallucis muscle. Evaluation of the distal tarsometatarsal insertions is difficult due to the deep location (Figure 21C) [49]. Moving the transducer further to the plantar surface, flexor hallucis longus and flexor digitorum longus tendons will be visualized (Figure 21D).

#### 4.3.3. Clinical Relevance

Dysfunction of the TP tendon is the most common cause of acquired flat foot. The majority of the pathologies are found at the segment across the medial malleolus or at the insertion on the navicular. Sprain or tear of the distal tendon slip may occasionally cause plantar foot pain. As the distal tendon slip is difficult to visualize (due to its deep location), magnetic resonance imaging may be needed for clarifying potential lesions [49].

### 4.4. Plantaris Tendon

#### 4.4.1. Anatomy

**Figure 22:**
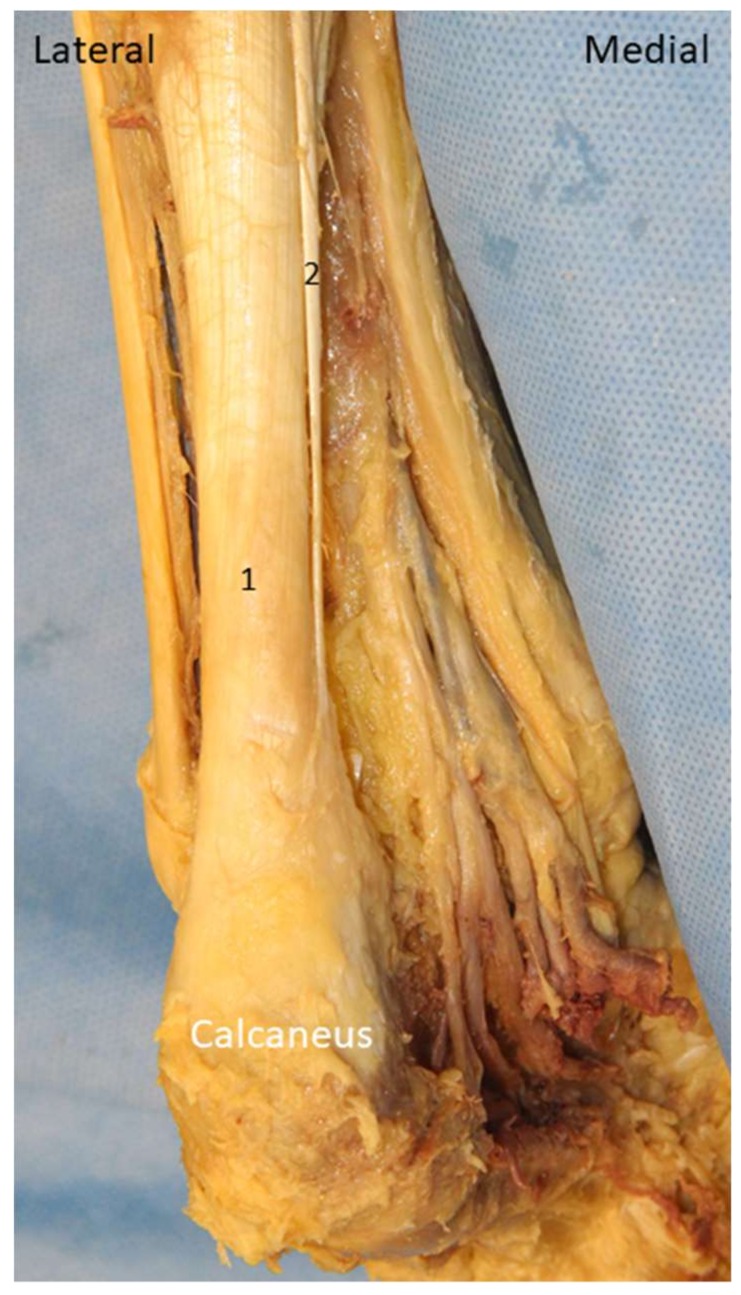
The plantaris tendon on the cadaveric model. 1: Achilles tendon; 2: plantaris tendon.

Plantaris is a small rudimentary muscle which is present in 91% of the general population [51]. It emerges from the posterior lateral supracondylar ridge of the femur and descends medial and oblique under the lateral head of gastrocnemius muscle [52]. The plantaris tendon fuses with the Achilles tendon distally and inserts on the calcaneus (Figure 22). 

#### 4.4.2. Scanning Technique

**Figure 23:**
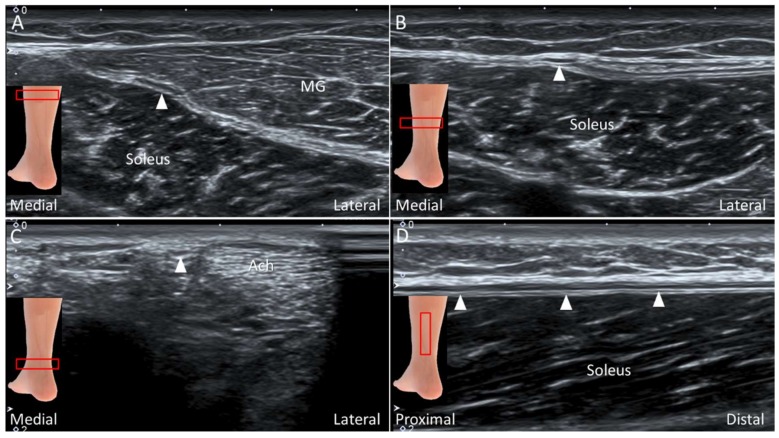
Imaging of the plantaris tendon (arrows) in the short-axis at the plane between medial gastrocnemius (MG) and soleus muscles (**A**), on the surface of soleus muscle adjacent to the MG tendon (**B**), medial to the Achilles tendon (Ach) (**C**)**,** and in the long-axis on the surface of soleus muscle (**D**).

The patient lies prone on the examination bed. The transducer is placed on the medial calf along the axial plane. The plantaris tendon is visualized as an oval shaped hyperechoic structure interposed between the medial gastrocnemius and soleus muscle (Figure 23A). The examiner can swap the transducer back and forth to trace the tendon. Distally, the medial gastrocnemius muscle tapers as a hyperechoic tendon lying on the surface of the soleus muscle, and the plantaris tendon is visualized medially to it (Figure 23B). More distally, gastrocnemius and soleus tendons form the Achilles tendon with the plantaris tendon located medially (Figure 23C). Multiple parallel hyperechogenic lines can be visualized in the tendon’s long axis (Figure 23D). 

#### 4.4.3. Clinical Relevance

An isolated lesion of the plantaris tendon is rare and can be easily misinterpreted as a medial gastrocnemius muscle tear (tennis leg) [53]. An isolated plantaris tendon rupture does not need surgical intervention and usually yields good prognosis after conservative management [52]. 

## 5. Nerves 

### 5.1. Medial Plantar Nerve (Over the Knot of Henry)

#### 5.1.1. Anatomy

**Figure 24:**
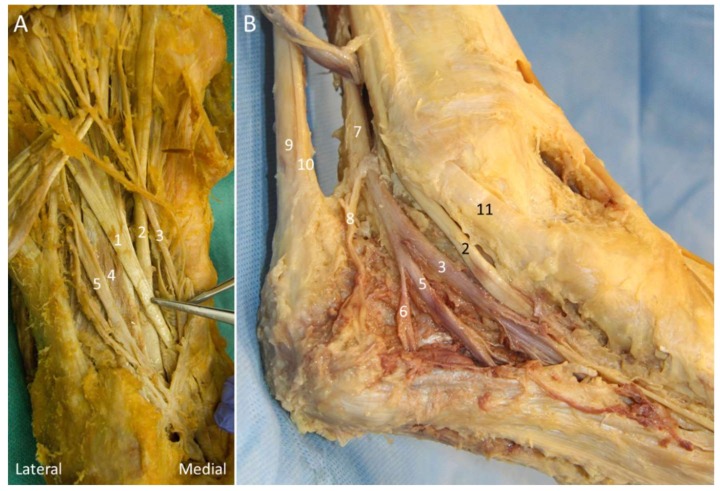
Cadaveric models for demonstrating the knot of Henry (**A**) and Baxter nerve (**B**). 1: Flexor digitorum longus tendon; 2: flexor hallucis longus tendon; 3: medial plantar nerve; 4: quadratus plantae muscle; 5: lateral plantar nerve; 6: Baxter nerve; 7: tibial nerve; 8: medial calcaneal nerve; 9: Achilles tendon; 10: plantaris tendon; 11: tibialis posterior tendon.

The knot of Henry corresponds to the decussation of the flexor hallucis longus and flexor digitorum longus tendons at the medial plantar mid-foot (at the navicular level). The flexor hallucis longus originates from the inferior two-thirds of the posterior fibula while the flexor digitorum longus originates from the posterior tibia and the fascia overlying the TP muscle [50]. These two tendons cross each other as they course distally (Figure 24A). The medial plantar nerve (MPN) is a terminal branch of the tibial nerve. After passing through the tarsal tunnel, the nerve traverses between the abductor hallucis and quadratus plantae muscles to reach the knot of Henry, and then it courses along the medial border of the flexor digitorum brevis muscle. 

#### 5.1.2. Scanning Technique

**Figure 25:**
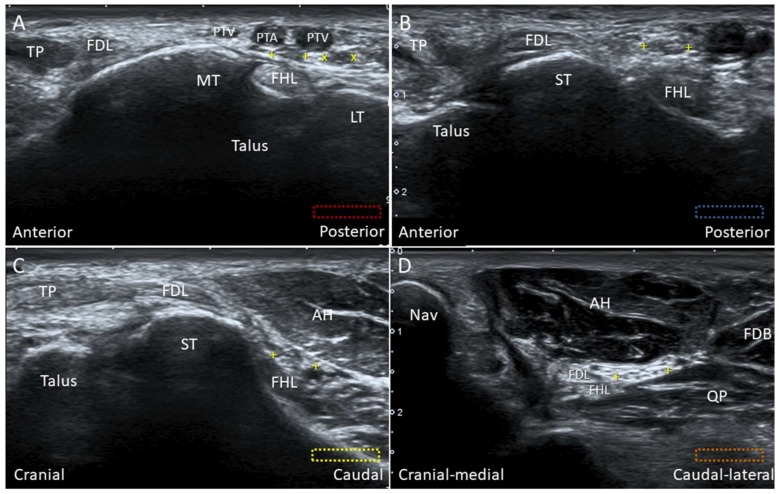
Imaging of the flexor hallucis longus tendon (FHL) at the tarsal tunnel (**A**), at the level posterior to the sustentaculum tali (ST) (**B**), at the level inferior to the ST (**C**)**,** and at the knot of Henry (**D**). The colored dashed rectangles are in accordance with the transducer positions in Figure 3A. “+ +”: medial plantar nerve; “x x”: lateral plantar nerve; AH: abductor hallucis muscle; FDB: flexor digitorum brevis muscle; FDL: flexor digitorum longus tendon; LT: lateral tubercle of the posterior talar process; MT: medial tubercle of the posterior talar process; Nav: navicular; PTA: posterior tibial artery; PTV: posterior tibial vein; QP: quadratus plantae muscle; TP: tibialis posterior tendon.

For scanning the knot of Henry, the transducer is initially placed behind the medial malleolus in the transverse plane. The flexor hallucis longus tendon is visualized between the medial and lateral tubercles of the posterior talar process (Figure 25A). Moving the transducer more distally, the flexor hallucis longus tendon is seen first besides (Figure 25B) and then under the sustentaculum tali (Figure 25C). The MPN is situated right on the flexor hallucis tendon. Sliding the transducer distally, the MPN moves to the plane between the quadratus plantae and abductor hallucis muscles (Figure 25D). 

#### 5.1.3. Clinical Relevance

The MPN can be entrapped adjacent to the knot of Henry secondary to repetitive eversion of the foot. This condition is common in runners, known as “jogger’s foot”, leading to pain on the medial side of the sole, radiating to the plantar aspect of the 1st three toes [54]. Tendinopathy of flexor hallucis and flexor digitorum longus tendons can cause MPN irritation. US-guided procedures such as hydrodissection (with 5% dextrose) of the nerve and surrounding tendons can be performed for symptom relief. 

### 5.2. Baxter Nerve 

#### 5.2.1. Anatomy

Baxter nerve, also known as the inferior calcaneal nerve and the first branch of the lateral planter nerve (LPN), is a small mixed nerve branching with a maximal diameter of 2 mm on average (Figure 24B) [55]. It gives off sensory innervation to the calcaneal periosteum and the LPL, and motor innervation to the quadratus plantae, flexor digitorum brevis, and abductor digiti minimi muscles. The Baxter nerve is reported to branch either from the LPN distal to the bifurcation point of the tibial nerve (or at the plantar foot) or from the tibial nerve (approximately 12%) [55,56]. The nerve courses between the abductor hallucis and quadratus plantae muscles initially and then between the flexor digitorum brevis and quadratus plantae muscles.

Three vertical fascial septa can be found in the sole: the medial septum (the dorsal extension of the medial border of the main plantar fascia), intermediate septum (the dorsal extension of the lateral border of the main plantar fascia), and the lateral septum (the dorsal extension of the lateral border of the LCPF) [57]. These septa form separated fibro-osseous tubes for nerve passage and serve as potential nerve entrapment points. The Baxter nerve passes through the medial and intermediate septa as it courses laterally towards the abductor digiti minimi muscle [10,58].

#### 5.2.2. Scanning Technique

**Figure 26:**
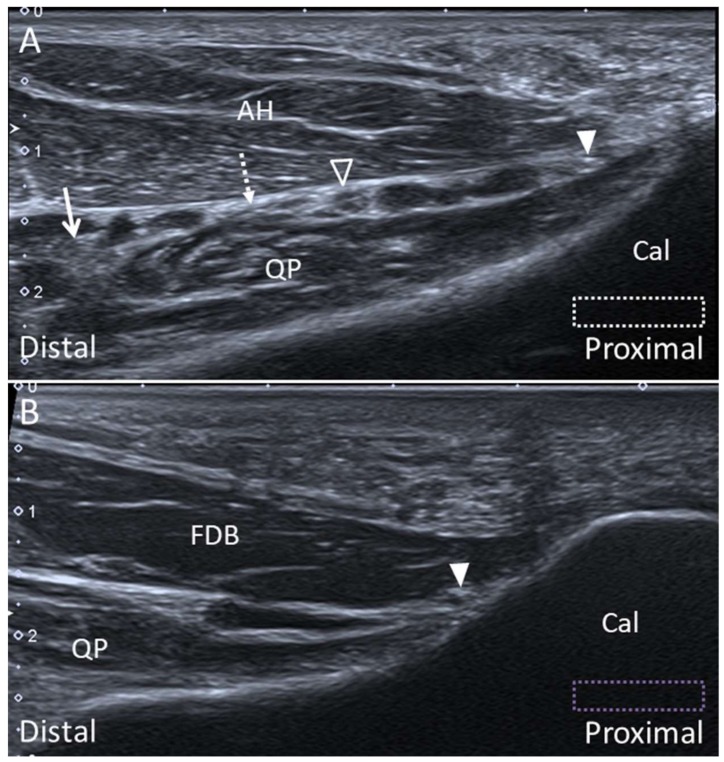
Ultrasound imaging of the Baxter nerve (arrowheads) between the abductor hallucis (AH) and the quadratus plantae (QP) muscles (**A**) and between the flexor digitorum brevis (FDB) and QP muscles (**B**). The colored dashed rectangles are in accordance with the transducer positions in Figure 3A,B. Lateral plantar nerve: void arrowhead; medial plantar nerve: arrow; medial septum: dotted arrow. Cal, calcaneus.

The transducer is placed in the transverse plane behind the medial malleolus to locate the tibial nerve. Moving the transducer to the plantar surface, the tibial nerve bifurcates to the MPN and LPN. The Baxter nerve can be seen emerging from the posterior margin of the LPN. The MPN, LPN, and Baxter nerve can be identified between the abductor hallucis and quadratus plantae muscles (Figure 26A). A hyperechoic connective tissue between the MPN and LPN can be visualized, representing the medial septum. At the plantar heel, the Baxter nerve appears as a hyperechoic small bubble between the flexor digitorum brevis and quadratus plantae muscles distal to the calcaneal tuberosity, when the transducer is placed in the sagittal plane (Figure 26B).

#### 5.2.3. Clinical Relevance

The Baxter nerve entrapment can be found in up to 20% of cases with chronic heel pain [55]. The nerve can potentially be entrapped at two sites: between the taut deep fascia of AH (the medial septum) and QP muscles, and distal to the edge of the medial calcaneal tuberosity in the presence of calcaneal spur or plantar fasciitis. The latter scenario is less likely to ensue when the Baxter nerve branches distally from the LPN at the plantar foot, as it remains far from the edge of the calcaneal tuberosity.

## 6. Conclusions

The ankle and foot harbor complex anatomical structures whereby US imaging can significantly facilitate the diagnostic and therapeutic algorithms in patients with relevant complaints. Of note, familiarization with the sonoanatomy is a prerequisite in this regard. Furthermore, the investigators should be aware of the potential pitfalls of using US imaging in exploring the ankle and foot, such as susceptibility to artifacts and lack of penetration for bony structures. Magnetic resonance imaging should be arranged if subcortical or subchondral lesions are suspected.

## Supplementary Materials

Supplementary materials can be found at https://www.mdpi.com/2075-4418/10/3/160/s1.
